# On the Mechanism of Action of Anti-Inflammatory Activity of Hypericin: An In Silico Study Pointing to the Relevance of Janus Kinases Inhibition

**DOI:** 10.3390/molecules23123058

**Published:** 2018-11-22

**Authors:** Luca Dellafiora, Gianni Galaverna, Gabriele Cruciani, Chiara Dall’Asta, Renato Bruni

**Affiliations:** 1 Department of Food and Drug, University of Parma, Area Parco delle Scienze 27/A, 43124 Parma, Italy; gianni.galaverna@unipr.it (G.G.); chiara.dallasta@unipr.it (C.D.A.); renato.bruni@unipr.it (R.B.); 2 Department of Chemistry, Biology and Biotechnology, University of Perugia, via Elce di Sotto, 8, 06123 Perugia, Italy; gabri@chemiome.chm.unipg.it

**Keywords:** *Hypericum perforatum*, hypericin, in silico pharmacodynamics, hypericin metabolites, anti-inflammatory activity, janus kinase 1

## Abstract

St. John’s Wort (*Hypericum perforatum* L.) flowers are commonly used in ethnomedical preparations with promising outcomes to treat inflammation both per os and by topical application. However, the underlying molecular mechanisms need to be described toward a rational, evidence-based, and reproducible use. For this purpose, the aptitude of the prominent *Hypericum* metabolite hypericin was assessed, along with that of its main congeners, to behave as an inhibitor of janus kinase 1, a relevant enzyme in inflammatory response. It was used a molecular modeling approach relying on docking simulations, pharmacophoric modeling, and molecular dynamics to estimate the capability of molecules to interact and persist within the enzyme pocket. Our results highlighted the capability of hypericin, and some of its analogues and metabolites, to behave as ATP-competitive inhibitor providing: (i) a likely mechanistic elucidation of anti-inflammatory activity of *H. perforatum* extracts containing hypericin and related compounds; and (ii) a rational-based prioritization of *H. perforatum* components to further characterize their actual effectiveness as anti-inflammatory agents.

## 1. Introduction

St. John’s Wort (*Hypericum perforatum* L.) flowers are commonly used in ethnomedical preparations to treat wounds and minor burns or to relieve a number of altered conditions, including depression and inflammation [1]. In particular, the effectiveness of *H. perforatum* preparations in treating mild-to-moderate depression has been recently recognized [2,3] and formulations derived from *H. perforatum* extracts are nowadays sold among the top-selling herb-based dietary and nutritional supplements [4,5,6]. *H. perforatum* flowers are also used to produce oils to treat topical inflammations [7] and the anti-inflammatory activity of extracts of various *Hypericum* species administered per os was described in animals [8,9,10,11]. However, a consensus has been achieved neither on the molecular mechanisms nor on the network of molecular targets involved in the anti-inflammatory activity, though critical for a more informed pharmacology of *H. perforatum* extracts and components thereof. In this regard, *H. perforatum* extracts typically include complex phytochemical mixtures and the pharmacologically relevant components still lack an adequate molecular profiling and characterization [12].

**Figure 1:**
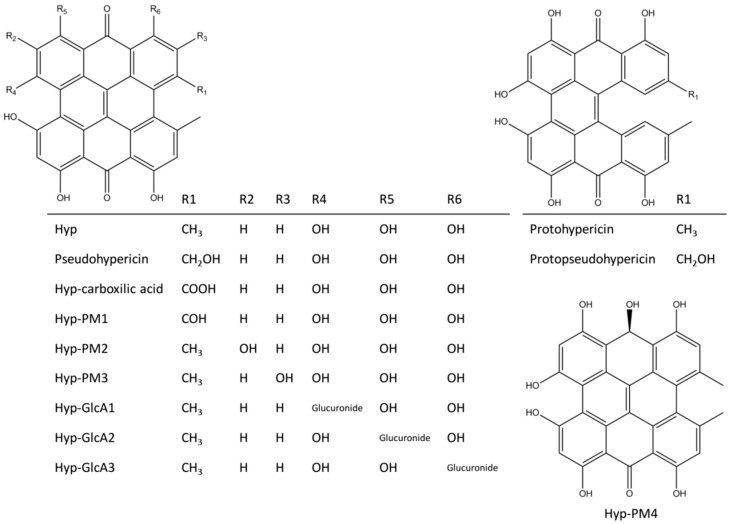
Chemical structures of Hyp and its analogues and human metabolites.

Among the compounds included in *H. perforatum* extracts, the class of napthodianthrones is one of the most prominent, with hypericin (Hyp, Figure 1) among the best characterized members [8]. A wide spectrum of desired bioactivities has been ascribed to Hyp and its congeners, including antibacterial and antiviral actions and antitumor properties upon light activation [13,14,15]. Anti-inflammatory activity has been found as well [16], pointing to the possibly relevant role of Hyp and related compounds in determining the anti-inflammatory activity of *Hypericum* preparations. Nevertheless, the understanding of the underlying mechanisms of action is still incomplete and further investigations are needed toward a more rational design and/or use of herb-based pharmacological interventions and food supplements. In this direction, the contributions of the naturally-occurring Hyp analogues and those of their metabolites circulating upon exposure need to be investigated with high priority.

Concerning the molecular basis of anti-inflammatory activity of Hyp and related *H. perforatum* components, the Janus Kinase-Signal Transducer and Activator of Transcription (JAK-STAT) system was found among the relevant pathways involved, and the reduction of JAK phosphorylation was proposed among the underlying mechanisms [17]. JAK is a family of tyrosine kinases constitutively associated to cytokine receptors acting as signal transducers downstream the cytokines receptors activation [18]. The binding of cognate ligands modifies receptors organization leading to auto-phosphorylation and subsequent activation of JAK. Activated JAKs cause phosphorylation of STAT resulting in the formation of STAT-STAT dimers, which are then moved to the nucleus where they may regulate the expression of specific genes involved in inflammatory response. The competitive binding with adenosine 5′-triphosphate (ATP) by small molecules proved to be an effective way to inhibit JAK activity, also to counteract inflammatory response [19]. As the down-regulation of pro-inflammatory cytokines was suggested as a possible mechanism involved in the *H. perforatum* extracts bioactivity, the existence of an interference exerted by Hyp and congeners on JAK-STAT may help elucidate St. John’s Wort properties [20]. Nevertheless, the series of molecular events and the biological targets underlying their possible effects on the JAK-STAT system still need to be described. 

In this framework, the aptitude of Hyp and some structural analogues to behave as JAK inhibitors was assessed in silico via molecular modeling, a technique that is getting more and more consensus in the high-throughput molecular characterization of pharmacologically relevant compounds [21,22,23]. Given the complex network of molecular events likely underlying the anti-inflammatory activity of *H. perforatum* components, the existence of possible direct effects of Hyp and congeners on JAKs were assessed on the basis of: (i) the involvement of JAK-STAT pathway previously described in the anti-inflammatory activity of mixtures containing Hyp congeners [17]; (ii) the relevance of JAK inhibition in anti-inflammatory activity [24]; (iii) the known activity of Hyp as kinases inhibitor, also in the lack of light activation [25]; (iv) evidences reporting the ATP-competitive inhibition of kinases by Hyp-related polyaromatic phenols [26]. In more detail, a structure-based approach relying on pharmacophoric modeling, docking studies and molecular dynamics was applied to study the capability of Hyp to interact and persist into the ATP pocket of JAK1, taken as reference for the JAK family. In addition, the capability of Hyp to comply with the ATP pocket was compared to that of some naturally occurring Hyp congeners and human metabolites (Figure 1) to identify additional forms that may contribute to JAKs inhibition in living organisms.

## 2. Results and Discussion

### 2.1. Pharmacophoric Analysis of the ATP Binding Site

**Figure 2:**
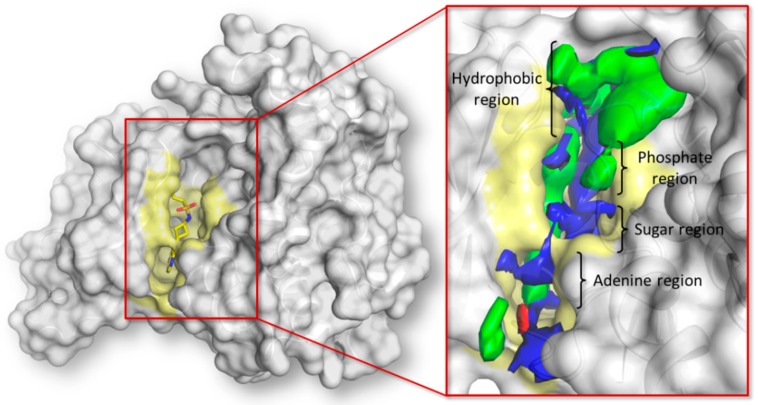
Representation of the JH1 domain of JAK1. The crystallographic structure (PDB ID 6BBU) [28] is represented in white surface while the co-crystallized ligand is represented in yellow sticks. The protein surface forming the ATP binding site is colored in pale yellow. The pharmacophoric fingerprint of the pocket is represented in the close-up where the green, red, and blue contours indicate the regions of the pocket sterically and energetically favorable to receive hydrophobic, hydrogen bond acceptor, and hydrogen bond donor groups respectively.

The JAK family groups four members (120–130 kDa; JAK1, JAK2, JAK3, and TYK2) and each protein comprises seven JAK homology domains (JH1–JH7). The C-terminal part (JH1) hosts the ATP-binding site and it is the catalytically active domain responsible for the physiological kinase activity [27]. This work focused on the JH1 domain of JAK1, taken as a reference for the JAK family. The ATP binding site consists in a solvent-exposed surface cleft formed by five different subsites as shown below: (i) adenine region, (ii) sugar region, (iii) phosphate binding region, (iv) hydrophobic pocket, and (v) hydrophobic channel. The pharmacophoric analysis of the ATP binding site showed an overall amphipathic environment with the polar space mainly able to receive hydrogen bond donors and a limited capability to receive hydrogen bond acceptor groups (Figure 2).

### 2.2. Training and Validation Procedures

The procedural reliability was assessed by challenging the JAK1 model with a training set including molecules with known binding architectures and strong experimentally proved inhibitory activity against JAK1 (i.e., true inhibitors) and virtual decoys, which are molecules sharing analogies with true inhibitors but supposed to be inactive and/or unable to bind the ATP binding site (further details are reported in Section 3.1). 

**Figure 3:**
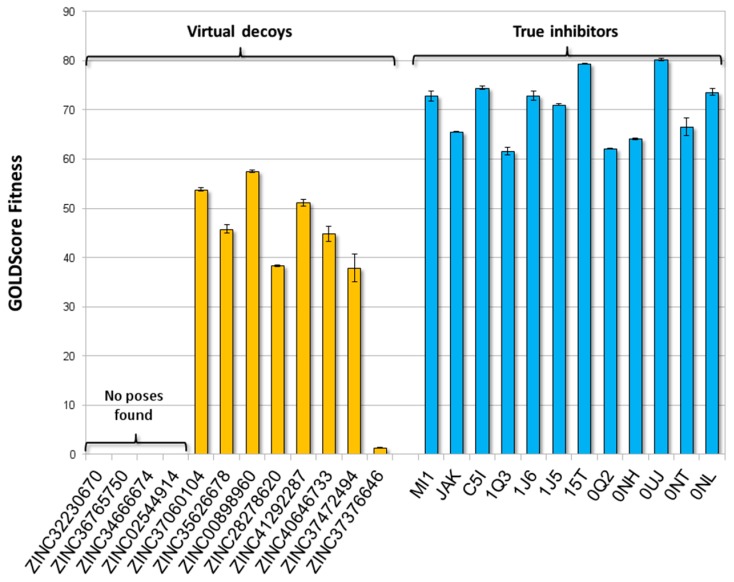
Docking results of training set. True inhibitors recorded scores significantly higher than those of decoys (*p* < 0.05; according to Games-Howell post hoc).

As shown in Figure 3, the whole subset of true inhibitors recorded scores significantly higher than those of decoys (*p* < 0.05; according to Games-Howell post hoc), although scores were not proportional to the experimental *K_i_* values reported in the literature (Table S1, Supporting Information). Concerning decoys, no poses were generated for ZINC36765750, ZINC34666674, and ZINC02544914, while ZINC32230670 was considered unable to stably fit the pocket due to the low score (26.0 units) and high coefficient of variation (60%) (see Section 3.4 for more details). Concerning the calculation of binding poses, the strong agreement between the computed and crystallographic poses of true inhibitors proved the procedure reliability in predicting the binding architecture of ligands (Figure S3, Supporting information).

On the basis of these results, the procedure reliably discriminated true inhibitors from decoys, confirming that the calculation of molecules fitting at the ATP-binding site could be used to qualitatively estimate their inhibitory activity. In addition, according to the precautionary principle and to reduce the occurrence of false negatives, the highest value recorded among decoys (i.e., 57.5 units, ZINC00898960) was set as threshold to estimate the activity of unknown compounds. Therefore, any compound recording a score significantly above the threshold was considered able to positively interact with the ATP-binding site and to possibly inhibit the enzyme.

**Figure 4:**
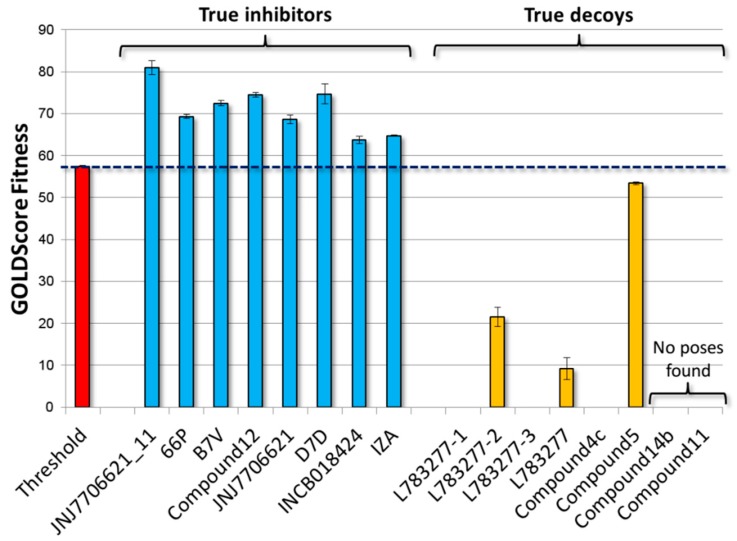
Docking results of validation set. True inhibitors recorded scores significantly higher than those of true decoys (*p* < 0.05; according to Games-Howell post hoc).

Afterwards, both the procedure and threshold were validated challenging the model with a validation set including true inhibitors and true decoys. The all set of true inhibitors, but not that of true decoys, recorded scores significantly above the threshold (*p* < 0.05; according to Games-Howell post hoc test) (Figure 4 and Table S2, Supporting Information). In particular, concerning true decoys, no poses were found for Compound **11**, while Compound **14b** was considered unable to stably fit the pocket due to the low score (14.0 units) and the high coefficient of variation (74%) (see Section 3.4 for more details). Notably, the set encompassed a wide range of molecular weight, volume, and number of hydrogen bond donors and acceptors, including some either comparable to or higher than Hyp. Their proper classification ultimately validated both the procedure and the threshold reliability excluding the existence of artificial enrichments possibly depending on molecular features (see Section 3 for further details). Nonetheless, the inclusion of both ligands and decoys structurally closer to Hyp, as soon as they will be characterized, might refine the threshold further strengthening model reliability.

On the basis of these results, assessing in silico the capability of small molecules to fit the ATP binding pocket proved to be an effective and reliable analytical approach to estimate their inhibitory activity against JAK1. Therefore, the procedure could be successfully applied to qualitatively estimate the inhibitory activity of Hyp and its analogues and metabolites.

### 2.3. Results of Hypericin

**Figure 5:**
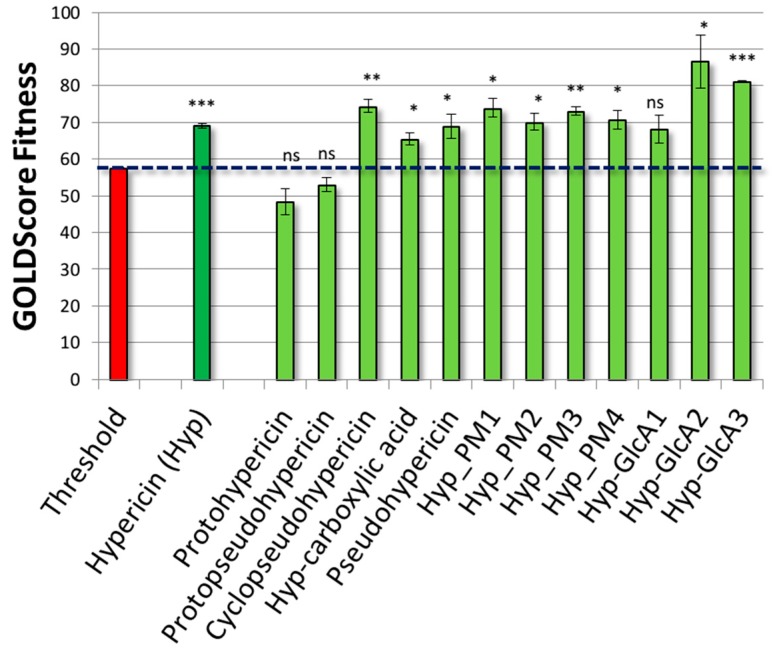
Docking results of Hyp, its analogues and metabolites. Significance to threshold value according to Games-Howell *post hoc* test: * *p* < 0.05, ** *p* < 0.01, *** *p* < 0.001, ns not significant.

**Figure 6:**
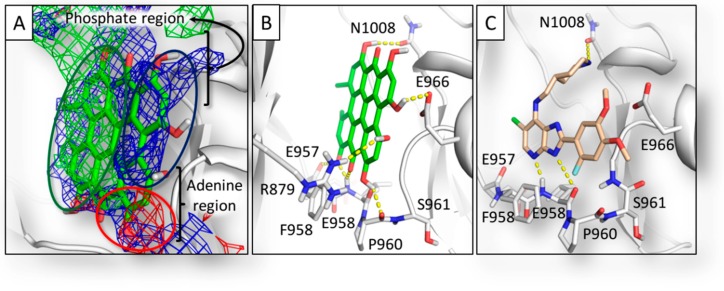
Binding architecture of Hyp. Protein is represented in white cartoon, while ligands and residues involved in polar contacts are represented in sticks. Yellow dotted lines represent polar contacts. (**A**) Hyp docked within the pharmacophoric space of binding site. Green, red, and blue meshes indicate regions sterically and energetically favorable to receive hydrophobic, hydrogen bond acceptor, and hydrogen bond donor groups respectively. Green, blue, and red rings indicate the pharmacophoric match of Hyp within the space suitable to receive hydrophobic, hydrogen bond acceptor and hydrogen bond donor groups, respectively. (**B**) Computed binding pose of Hyp. (**C**) Crystallographic binding pose of 5GJ [29].

Once validated, the model was challenged with Hyp. As shown in Figure 5, Hyp recorded a score significantly above the threshold and, therefore, it was considered able to positively interact with the ATP binding pocket. As shown in Figure 6, Hyp fitted the pharmacophoric space of the pocket (Figure 6A) where it docked the adenosine region, retracing in such a way the mode of binding of the known inhibitor 6-chloro-2-(2-fluoro-4,5-dimethoxyphenyl)-*N*-(piperidin-4-ylmethyl)-3H-imidazo[4–b]pyridin-7-amine (PDB code 5GJ) [29], and protruding toward the phosphate region (Figure 6B,C).

**Figure 7:**
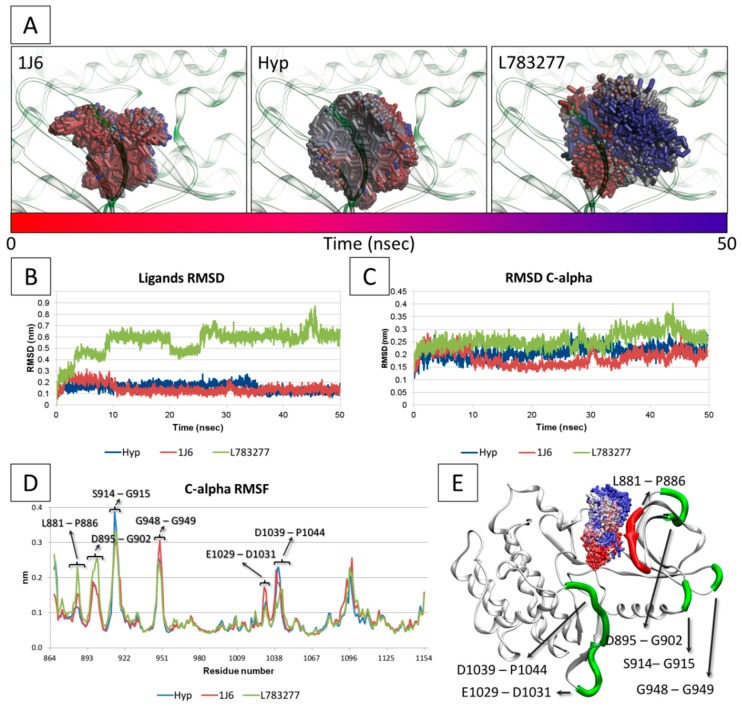
Conformational changes of JAK1 in complex with the true inhibitor 1J6, Hyp or the true decoy L783277. (**A**) Time-step representation of the trajectories of 1J6, Hyp and L783277. The from-red-to-blue color switch indicates the stepwise changes of ligand coordinates along the MD simulation. L783277 showed the tendency to get out the binding site as the blue-colored coordinates headed outward the site boundaries. Conversely, Hyp and 1J6 persisted into the binding site during the all MD simulations. (**B**) RMSD plot of 1J6, Hyp and L783277. (**C**) RMSD plot of protein C-alpha in complex with 1J6, Hyp or L783277. (**D**) RMSF plot of protein residues. (**E**) Graphical representation of protein in complex with L783277. Protein is represented in cartoon while L783277 is shown in stick using the red-to-blue time-step coloring. The regions showing differential mobility are thickened and colored in green, while the differentially mobile region forming the binding site is colored in red.

In addition, the capability of Hyp to persist into the ATP-binding site was assessed using MD simulations. The dynamic of interaction were compared to that of the strong inhibitor 1J6, which had the lowest K_i_ value among those included in the training set, and the true decoy L78327. The trajectory of ligands and the root-mean-square analysis (RMSD) of protein C-alpha and ligands’ atomic coordinates were analyzed to measure the structural stability of complexes. As shown in Figure 7, the true inhibitor 1J6 was found persisting into the ATP binding site during the whole MD simulation (Figure 7A). Moreover, RMSD of ligand coordinates showed low and stable values confirming its structural stability into the pocket (Figure 7B). Conversely, the true decoy L78327 clearly showed a trajectory outward the binding pocket, in agreement with its geometrical instability pointed out by the progressive increase of RMSD values (Figure 7A,B). It is important to note that Hyp showed a persistence into the binding site and RMSD values similar to that of the true inhibitor 1J6 (Figure 7A,B) pointing to its capability to fit and persist into the ATP binding site.

The RMSD analysis of protein C-alpha revealed the overall structural stability of protein along the MD simulation regardless the ligand bound. However, the complex with L78327 showed a RMSD progression slightly higher than that with Hyp and 1J6, which were instead quite similar to each other further supporting the capability of Hyp to stably dock the pocket (Figure 7C). 

The root-mean-square fluctuation (RMSF) analysis of protein C-alpha in the three protein-ligand complexes was performed to measure locally the mobility of protein residues. As shown in Figure 7D,E, comparable fluctuations were found in the three complexes, except for six regions depending on the ligand bound (i.e., the regions L881-P886, D895-G902, S914-G915, G948-G949, E1029-D1031, and D1039-P1044). Interestingly, L881-P886 is a part of the loop forming the binding site in direct contact with ligands. In that region, the protein in complex with L78327 showed the highest mobility, consistently with the need to open the binding site to allow the outward trajectory of L78327. It is worth to mention that in this region the Hyp-protein complex showed values lower than those of the 1J6-protein complex. This finding may indicate the capability of Hyp to effectively stabilize the region forming the binding site, further pointing to its capability to fit the ATP binding site and persist therein. 

Taken together, these results gave a molecular insight pointing to the possible anti-inflammatory properties of Hyp via JAK1 inhibition. The competition with ATP was proposed as a likely mechanism of action. Notably, considering the promiscuity of ATP-competitive inhibitors, Hyp might have a broader activity inhibiting also other members of the JAK family. These results may be of high relevance to the rational prioritization of future studies aimed at characterizing the mechanisms and mode of action of Hyp and Hyp-containing extracts, as well as in developing pharmacologically active molecules using Hyp as scaffold.

### 2.4. Results of Hypericin Analogues and Metabolites

**Figure 8:**
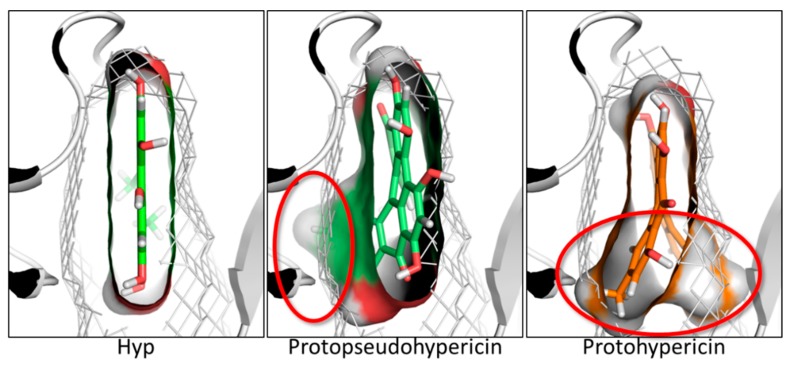
Comparison between the docking pose of Hyp, protopseudohypericin and protohypericin. Protein is represented in cartoon, the shape of the binding site is traced in mesh, while ligands are represented in cut surfaces and sticks. Red rings indicate the region of molecules not matching the shape of the binding site and reducing the fitting therein.

Hyp can occur in planta together with diverse ratios of several structural congeners that are likely to have combined effects in modulating the overall anti-inflammatory activity of formulations containing Hyp and related compounds. The precursors protohypericin and protopseudohypericin and the analogues pseudohypericin, cyclopseudohypericin, and Hyp-carboxylic acid are the most abundant [30]. Therefore, their possible relevance in the JAK inhibition was assessed. Protohypericin and protopseudohypericin were found unable to fit the pocket recording scores below the threshold (Figure 6). Their scores dropped due to the loss of planarity (Figure 8), even though both complied with the distribution of polar and hydrophobic space of the pocket. Accordingly, for them a lower capability to interact with the ATP binding site and to competitively inhibit enzymatic activity could be hypothesized. Conversely, pseudohypericin, cyclopseudohypericin, and Hyp-carboxylic acid recorded scores above the threshold (Figure 6). They complied with the pharmacophoric space of pocket although they adopted an orientation slightly different than that of Hyp due to the steric restraints caused by scaffold modifications. However, all of them kept the main interactions showed by Hyp (Figure 6) with no supplementary interactions found. Taken together these results indicated that some but not all the Hyp analogues occurring in *H. perforatum* might be involved in the anti-inflammatory activity via JAK inhibition. Moreover, a combined effect is likely to occur. Notably, the loss of planarity was identified as an important structural feature preventing the pocket fitting.

To provide a more comprehensive evaluation, the analysis was extended to a set of phase I and phase II human Hyp metabolites, given the relevance of forms actually circulating upon exposure to understand both the dynamic and kinetic aspects of pharmacologically active compounds [31]. Notably, the human metabolism of Hyp is still a largely overlooked issue, hindering the overall understanding of Hyp pharmacology. It is believed that Hyp does not undergo a massive metabolism, although evidences of phase I metabolism and phase II glucuronidation have been collected [32]. In this work, the plausible structures of six Hyp phase I metabolites and three isomers of Hyp-glucuronides (Hyp-GlcAs) were predicted in silico (Figure 1) to estimate their fitting into the ATP binding site. The set of phase I metabolites included the already identified structures of pseudohypericin and Hyp-carboxilate, derived from the phase I oxidative metabolism, along with one reduced metabolite (Hyp-PM4) and three oxidized metabolites (Hyp-PM1, Hyp-PM2 and Hyp-PM3) never identified before. All these metabolites fitted the ATP pocket and a certain degree of inhibitory activity could be expected accordingly. In particular, Hyp-PM2 and Hyp-PM3 retraced the binding architecture of Hyp. Notably, Hyp-PM2 used the additional hydroxyl group to engage R879 with a supplementary hydrogen bond (Figure 6). The binding pose of Hyp-PM1 retraced those of pseudohypericin, cyclopseudohypericin and Hyp-carboxilic acid with no additional interactions found. Conversely, the modification of Hyp-PM4 (Figure 1) allowed the formation of an additional hydrogen bond with the protein backbone (carbonyl oxygen of G1020), while keeping the same pocket occupancy showed by Hyp-PM1. These results suggested that metabolism of Hyp might have an overall limited capability to hamper the interaction with JAK1, highlighting the importance of better describing its pharmacokinetics toward a more informed understanding of the effects in living organisms.

## 3. Materials and Methods 

### 3.1. Design of Training and Validation Sets 

As previously described [33], the definition of a training set (Table S1, Figure S1) aimed at: (i) checking the procedure reliability to distinguish true inhibitors (i.e., experimentally determined JAK inhibitors binding at the ATP site) from decoys (i.e., compound unable to bind ATP site and/or unable to inhibit JAK); (ii) defining a significant score threshold to estimate the activity of uncharacterized molecules. RCSB PDB databank (https://www.rcsb.org/) [34] sourced the subset of true inhibitors and all the available ATP-competitive inhibitors of JAK1 with known K_i_ values were included (12 compounds, Table S1; last database access 27 March 2018). The subset of decoys was derived using the generation of virtual decoys, on the basis of previous results [35]. Virtual decoys were obtained querying the public available ZINC database (http://zinc.docking.org/) [36] with DecoyFinder (version 2.0) [37] using the all set of true inhibitors as input. The “all standard” ZINC subset was queried (last database access 27 March 2018) using two subsequent searches. The first search identified 50 decoys for each ligand with 2 units set as the maximum standard deviation from true inhibitors. The output underwent a second search based on physicochemical molecular descriptors to identify one decoy for each true inhibitor. Parameters were set as follows: Tanimoto coefficient threshold between ligands and decoys: 0.3; molecular weight range: ±40 Da; number of hydrogen bond donors and acceptors: ±3; number of rotatable bonds: ±3.

The validation set (Table S2, Figure S2) grouped true inhibitors and true decoys and included molecules derived from the literature with experimentally-determined inhibitory activity against JAK1. Specifically, the set grouped molecules spanning over wide ranges of molecular weight, volume and number of hydrogen bond donors/acceptors, including some decoys either with values comparable to or higher than Hyp, to check the lack of artificial enrichments which may due to the molecular features (commonly, larger the molecule, higher the score and easier the enrichment of bulky molecules) [38].

### 3.2. Model Preparation for Docking Simulations

The model of human JAK1 was derived from the crystallographic structure deposited in the RCSB PDB databank (https://www.rcsb.org/) [34] with ID codes 6BBU [28]. All the 3D coordinates of crystallographic ligands (i.e., true inhibitors) were retrieved from RCSB PDB databank (https://www.rcsb.org/) [34], while those of Hyp, protohypericin, and protopseudohypericin were retrieved from PubChem database (National Center for Biotechnology Information, https://pubchem.ncbi.nlm.nih.gov/; accessed March 2018) [39]. The 3D chemical structures of the other ligands were drawn using the Sybyl software, version 8.1 (Princeton, NJ, USA) (www.certara.com). Before proceeding with calculations, the consistency of atom and bond types of protein and ligands was checked with the Sybyl software (Princeton, NJ, USA) as described previously [40]. Co-crystallized ligand occupying the binding site was removed to prepare the model for docking simulations. In addition, the comparison of the crystallographic structures of human JAK1 available so far (last database access 27 March 2018) revealed that the water molecules number 4133, 4153, 4155, and 4183 were systematically conserved and therefore they were kept in the model.

### 3.3. Pharmacophoric Modeling

The binding site of JAK was defined using the Flapsite tool of the FLAP software (Borehamwood, UK) (www.moldiscovery.com) while the GRID algorithm was used to investigate the corresponding pharmacophoric space [41,42]. The DRY probe was used to describe potential hydrophobic interactions, while the sp2 carbonyl oxygen (O) and the neutral flat amino (N1) probes were used to describe the hydrogen bond acceptor and donor capacity of the target, respectively.

### 3.4. Docking Simulations

GOLD (Genetic Optimization for Ligand Docking) software (Cambridge, UK) was chosen to perform docking studies as it already succeeded in computing ligand-target interactions and estimating the inhibitory activity of ligands against enzymes [43,44,45,46]. The occupancy of binding site was rewarded setting a 3 Å sphere at the center of pocket and the option to skip poses generation when docking is physically impossible was set. Software setting and docking protocol previously reported were used [47]. As an exception, the use of external scoring functions was omitted as the GOLD’s internal scoring function GOLDScore succeeded in analyzing the reference set of compounds (see Section 2.2 for further details). In addition, molecules showing a coefficient of variation higher than 30% were considered unable to find a stable binding architecture. Accordingly, they were considered unable to favorably bind the pocket.

### 3.5. Molecular Dynamic

Molecular dynamic (MD) simulations were done to investigate the permanence of Hyp into the ATP binding site in comparison to a true inhibitor (i.e., 1J6) [21] and a true decoy (i.e., L783277) [48] taken as reference. The binding poses calculated by docking simulation were used as input for MD. Notably, the model used for docking simulation showed some unresolved peripheral loops (residues 912–917 and 946–950). Therefore, the structural continuity was obtained by means of homology modeling using the software Modeller (version 9.19) (San Francisco, CA, USA) [49]. In particular, the primary structure of JAK1 was set as the query and the 3D JAK1 model used for docking simulation was set as the template to reconstitute loop continuity. MD simulations were performed using GROMACS (version 5.1.4) (Stockholm, Sweden) [50] with CHARMM27 all-atom force field parameters support [51]. All the ligands have been processed and parameterized with CHARMM27 all-atom force field using the SwissParam tool (http://www.swissparam.ch) [52]. Crystallographic waters kept in the docking studies were removed and protein-ligand complexes were solvated with SPCE waters in a cubic periodic boundary condition, and counter ions (Na^+^ and Cl^−^) were added to neutralize the system. Prior to MD simulation the systems were energetically minimized to avoid steric clashes and to correct improper geometries using the steepest descent algorithm with a maximum of 5000 steps. Afterwards, all the systems underwent isothermal (300 K, coupling time 2 psec) and isobaric (1 bar, coupling time 2 psec) 100 psec simulations before running 50 nsec simulations (300 K with a coupling time of 0.1 psec and 1 bar with a coupling time of 2.0 psec). 

### 3.6. Prediction of Metabolites

All the putative Hyp metabolites were computed by using MetaSite (Borehamwood, UK) (http://moldiscovery.com) [53] and Way2Drug (Moscow, Russia) (http://www.way2drug.com) [54] for phase I and phase II metabolism, respectively. In both predictions, human metabolism was accounted and only the first generation of metabolites was collected. 

### 3.7. Statistical Analysis

Each docking run was performed in quadruplicate and data are expressed as the mean of at least three replicates ± standard deviation (SD). GOLD implements a genetic algorithm that may introduce variability in the results in a system-dependent manner possibly causing outliers. Therefore, outlier points were removed to a maximum of one for each run after being identified with the modified Thompson Tau Test, as already reported [55]. Data were statistically compared by one-way ANOVA (α = 0.05), using IBM SPSS Statistics for Linux, version 25 (IBM Corp., Armonk, NY, USA).

### 3.8. Graphical Representation

Marvin (version 18.25; ChemAxon; https://www.chemaxon.com) (Budapest, Hungary) was used for drawing and displaying two-dimensional chemical structures. Three-dimensional representations were obtained using PyMOL (The PyMOL Molecular Graphic System, Version 2.0.6 Schrödinger, LCC.) (New York, NY, USA) or VMD version 1.9.2 (http://www.ks.uiuc.edu/Research/vmd/) (Urbana and Champaign, Illinois, US) [56].

## 4. Conclusions

The present work dealt with the early mechanistic analysis of Hyp and related compounds to elucidate possible mechanisms underlying their anti-inflammatory action, which has been found a promising ancillary activity along with the antiviral, antibacterial, and antitumor properties. In this framework, JAK1 was identified among the network of targets reasonably involved, even though effects on the other JAK family members cannot be excluded given the low intra-family specificity of JAKs inhibitors. In more detail, the computational assessment of ligands interaction at the ATP binding site of JAK1 could be reliably used to qualitatively estimate the inhibitory activity of Hyp and related compounds. The molecular modeling study showed that Hyp and some analogues are likely to directly contact and possibly inhibit JAK1 providing: (i) a mechanistic rationale explaining, at least in part, the anti-inflammatory activity of *H. perforatum* extracts pointing to the likely inhibition of JAK1 by Hyp and some analogues; and (ii) an evidence-based top-ranking of JAKs as high-priority candidate for further characterization studies toward a more informed pharmacology of Hyp and other *H. perforatum* components. 

Moreover, the analysis of Hyp congeners showed that pseudohypericin, cyclopseudohypericin, and Hyp-carboxylic acid and some Hyp human metabolites may be able to interact with the pocket possibly contributing to inhibit the enzyme. The molecule planarity was identified as a structural prerequisite to bury into the surface cleft, suggesting that such feature should be maintained when using Hyp as a lead compound for future drug development. These data also suggest that the overall anti-inflammatory activity of *H. perforatum* extracts could be a consequence of the summative effect of complex mixtures of compounds, in agreement with previous studies [57], wherein Hyp and related compounds are likely to have an important role. In addition, some phase-I and phase-II Hyp metabolites may keep the capability to interact too, possibly playing a relevant role in the overall effect in living organisms. This evidence should be taken into account in designing future pharmacological investigations and possible drug development operations on Hyp and related compounds for anti-inflammatory purposes.

## Supplementary Materials

The following are available online. **Figure S1.** Chemical structures of compounds included in the training set; **Figure S2.** Chemical structures of compounds included in the validation set; **Figure S3.** Comparison between calculated and crystallographic pose of true inhibitors included in the training set; **Table S1.** Experimental and calculated values of true inhibitors included in the training set; **Table S2.** Experimental and calculated values of molecules included in the validation set.
