# Supplementary material for: On the Mechanism of Action of Anti-Inflammatory Activity of Hypericin: An In Silico Study Pointing to the Relevance of Janus Kinases Inhibition
Source: Molecules. 2018 Nov 22;23(12):3058. doi: 10.3390/molecules23123058 (PMC6321526; doi:10.3390/molecules23123058)
Supplement: Supplementary file 1 [file molecules-23-03058-s001.pdf]

Supporting Information

On the mechanism of action underlying the anti-inflammatory activity of hypericin: an *in silico* study pointing to the relevance of janus kinases inhibition

Luca Dellafiora [1]*, Gianni Galaverna [1], Gabriele Cruciani [2], Chiara Dall'Asta [1], Renato Bruni [1]

[1] Department of Food and Drug, University of Parma, Area Parco delle Scienze 27/A, 43124 Parma, Italy

[2] Department of Chemistry, Biology and Biotechnology, University of Perugia, via Elce di Sotto, 8, 06123 Perugia, Italy

* Correspondence to: Luca Dellafiora, Department of Food and Drug, University of Parma, Area Parco delle Scienze 27/A, 43124 Parma, Italy. Email: luca.dellafiora@unipr.it. Phone: +390521906196

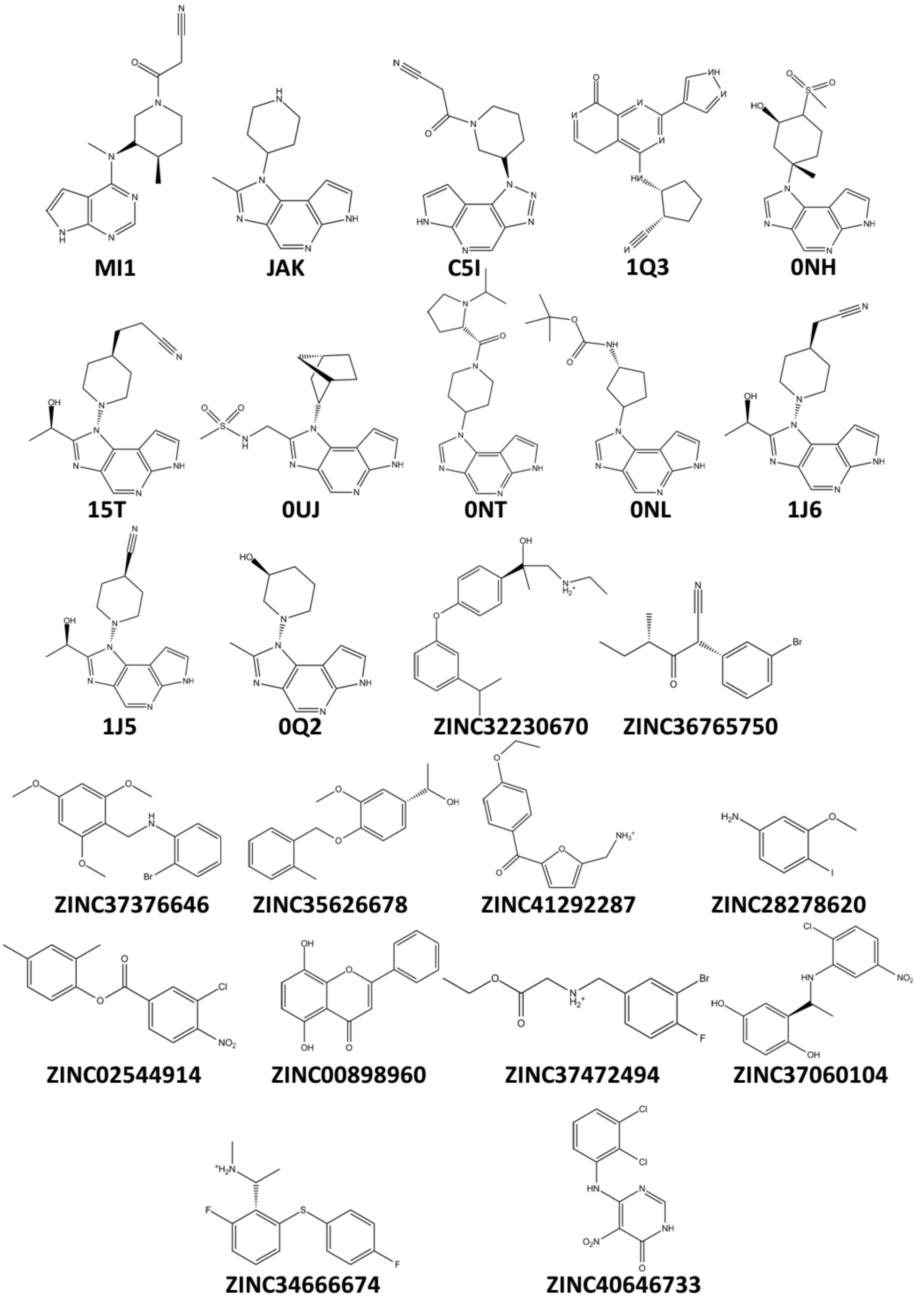

Figure S1. Chemical structures of compounds included in the training set.

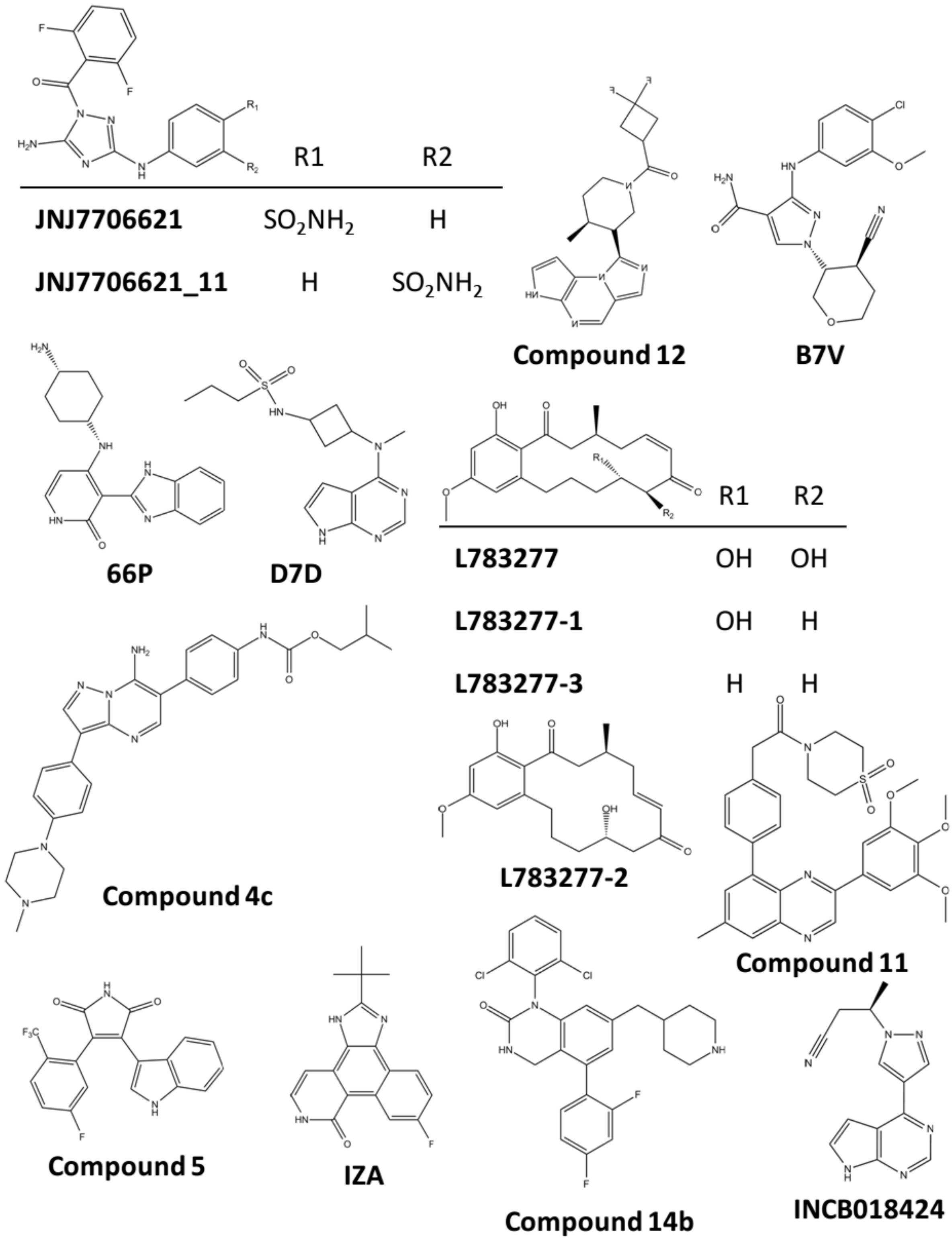

Figure S2. Chemical structures of compounds included in the validation set.

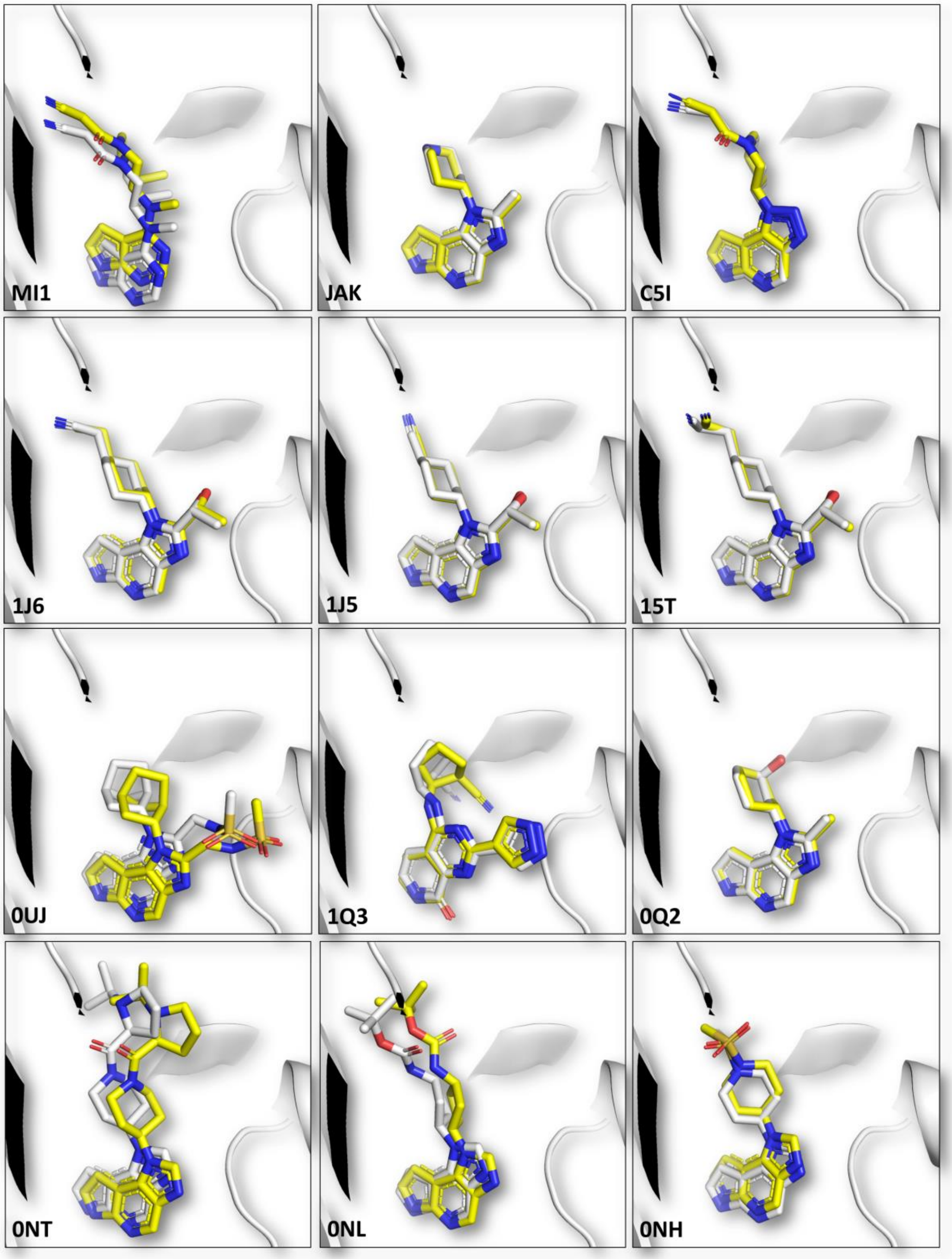

Figure S3. Comparison between calculated (yellow) and crystallographic (white) pose of true inhibitors included in the training set.

The subset of true inhibitors belonging to the training set was retrieved from the RCSB PDB databank (https://www.rcsb.org/). Experimental activities and computed scores are reported below (Table S1).

Table S1. Experimental and calculated values of true inhibitors included in the training set

Compound (PDB ID)	Experimental K_i (nM) [a]	Computational Scores
MI1	0.7	78.8 ± 1.0
JAK	10	65.6 ± 0.0
C5I	1.5	74.5 ± 0.4
1Q3	2	61.7 ± 0.8
1J6	0.1	72.9 ± 1.0
1J5	1.9	71.1 ± 0.2
15T	0.3	79.3 ± 0.1
0Q2	1.8	62.1 ± 0.1
0NH	1.7	64.1 ± 0.1
0UJ	0.2	80.2 ± 0.2
0NT	22	66.5 ± 1.8
0NL	0.9	73.6 ± 0.7

[a] according to the external ligands annotation reported in the RCSB PDB databank (https://www.rcsb.org/; last database access 27th March 2018)

Table S2. Experimental and calculated values of molecules included in the validation set

Compound	Experimental activity	Computational Scores	Computed activity [a]
66P	Active [b]	69.4 ± 0.5	Yes
B7V	Active [b]	72.5 ± 0.7	Yes
D7D	Active [b]	74.7 ± 2.4	Yes
IZA	Active [b]	64.8 ± 0.2	Yes
JNJ7706621_11	Active [c]	81.0 ± 1.6	Yes
Compound 12	Active [d]	74.5 ± 0.5	Yes
JNJ7706621	Active [c]	68.6 ± 1.1	Yes
INCB018424	Active [c]	63.7 ± 0.9	Yes
L783277	Inactive [e]	9.2 ± 2.7	No
L783277-1	Inactive [e]	0	No
L783277-2	Inactive [e]	21.6 ± 2.3	No
L783277-3	Inactive [e]	0	No
Compound 4c	Inactive [f]	0	No
Compound 5	Inactive [g]	53.4 ± 0.3	No
Compound 14b	Inactive [h]	No poses found	No
Compound 11	Inactive [i]		No

[a] the threshold above which compounds were considered able to positively interact with the pocket was set at 57.5 units

[b] according to the external ligands annotation reported in the RCSB PDB databank (https://www.rcsb.org/; last database access 27th March 2018)

[c] Malerich et al., 2010

[d] Norman, 2012

[e] Liniger et al., 2011

[f] Gommermann et al., 2010

[g] Thoma et al., 2011

[h] Stelmach et al., 2003

[i] Pissot-Soldermann et al., 2010

References not included in the main manuscript

Gommermann N, Buehlmayer P, von Matt A, Breitenstein W, Masuya K, Pirard B, Furet P, Cowan-Jacob SW, Weckbecker G. New pyrazolo[1,5a]pyrimidines as orally active inhibitors of Lck. Bioorg Med Chem Lett. 2010. 20 (12), 3628-3631.

Liniger M, Neuhaus C, Hofmann T, Fransioli-Ignazio L, Jordi M, Drueckes P, Trappe J, Fabbro D, Altmann KH. Kinase inhibition by deoxy analogues of the resorcylic lactone L-783277. ACS Med Chem Lett. 2010. 2 (1), 22-27.

Malerich JP, Lam JS, Hart B, Fine RM, Klebansky B, Tanga MJ, D'Andrea A. Diamino-1,2,4-triazole derivatives are selective inhibitors of TYK2 and JAK1 over JAK2 and JAK3. Bioorg Med Chem Lett. 2010. 20 (24), 7454-7457.

Norman P. Selective JAK1 inhibitor and selective Tyk2 inhibitor patents. Expert Opin Ther Pat. 2012. 22 (10), 1233-1249.

Pissot-Soldermann C, Gerspacher M, Furet P, Gaul C, Holzer P, McCarthy C, Radimerski T, Regnier CH, Baffert F, Drueckes P, Tavares GA, Vangrevelinghe E, Blasco F, Ottaviani G, Ossola F, Scesa J, Reetz J. Discovery and SAR of potent, orally available 2,8-diaryl-quinoxalines as a new class of JAK2 inhibitors. Bioorg Med Chem Lett. 2010. 20 (8), 2609-2613.

Stelmach JE, Liu L, Patel SB, Pivnichny JV, Scapin G, Singh S, Hop CE, Wang Z, Strauss JR, Cameron PM, Nichols EA, O'Keefe SJ, O'Neill EA, Schmatz DM, Schwartz CD, Thompson CM, Zaller DM, Doherty JB. Design and synthesis of potent, orally bioavailable dihydroquinazolinone inhibitors of p38 MAP kinase. Bioorg Med Chem Lett. 2003. 13 (2), 277-280.

Thoma G, Nuninger F, Falchetto R, Hermes E, Tavares GA, Vangrevelinghe E, Zerwes HG.

Identification of a potent Janus kinase 3 inhibitor with high selectivity within the Janus kinase family. J Med Chem. 2011. 54 (1), 284-288
